# Supplementary material for: Temporally restricted activation of IFNβ signaling underlies response to immune checkpoint therapy in mice
Source: Nat Commun. 2022 Aug 19;13:4895. doi: 10.1038/s41467-022-32567-8 (PMC9390963; doi:10.1038/s41467-022-32567-8)
Supplement: Supplementary file 1 — Supplementary Information [file 41467_2022_32567_MOESM1_ESM.pdf]

SUPPLEMENTARY FIGURES

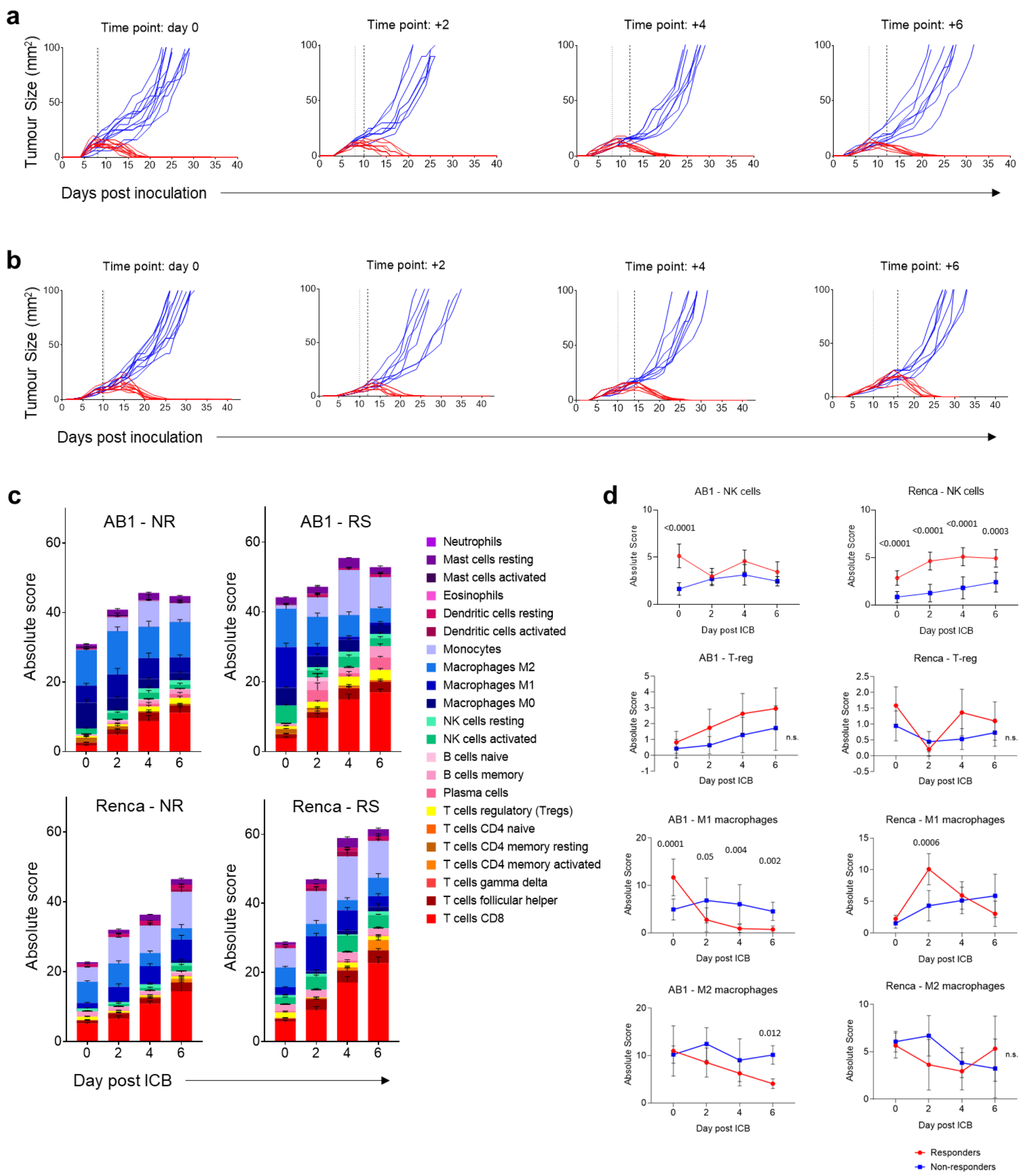

Supplementary Figure 1. Growth curves of remaining tumors left in situ after surgical removal of the contralateral AB1 and Renca tumors. Mice bearing contralateral tumors had one tumor removed for RNA sequencing, with the remaining tumor monitored for response to ICB. (**a**) AB1 tumors were treated with ICB on day 8, and one tumor excised 1 hour, 2, 4, or 6 days after. (**b**) Renca tumors were treated with ICB on day 10, and one tumor excised 1 hour, 2, 4, or 6 days after. Tumors were categorized as responders (red lines) or non-responders (blue lines). Dotted line = beginning of ICB treatment. Dashed line = day of surgical removal of tumor on right side. (**c**) CIBERSORTx cell deconvolution analysis of responder (RS) and non-responder (NR) tumors

over time, showing all cell subsets. Error bars represent SD.(**d**) Dynamics of NK cells, T-reg, M1 macrophages and M2 macrophages in responders (red) and non-responders (blue) over time in the AB1 (left) and Renca (right) models. Data are presented as mean values +/- SD. *p ≤ 0.05, **p ≤ 0.01, ****p ≤ 0.001 , ****p < 0.0001 from two-way ANOVA with Tukey's multiple comparisons test. N = 8-12 biologically independent samples per group (144 total). Source data are provided in the Source Data file.

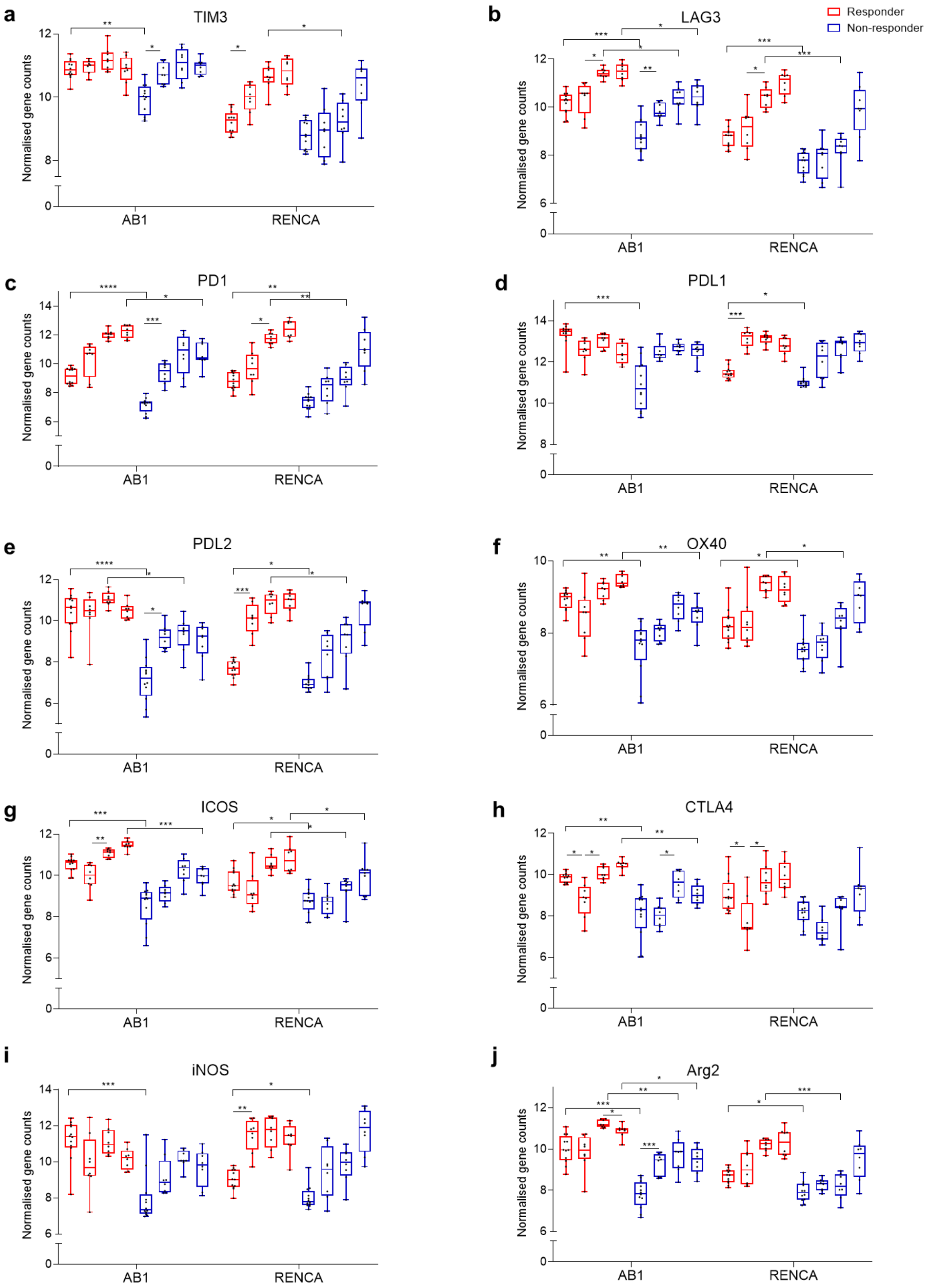

Supplementary Figure 2. Expression of immune checkpoints over the course of ICB from the bulk RNAseq data . Gene expression in responders (red) and non-responders (blue) over time (day 0, 2, 4, 6) in the AB1 and Renca models. Gene expression for TIM3 (a), LAG3 (b), PD1 (c), PDL1 (d), PDL2 (e), OX40 (f), ICOS (g), CTLA4 (h), iNOS (i) and Arg2 (j). p ≤ 0.05, **p ≤ 0.01, ****p ≤ 0.001 , ****p < 0.0001 from two-way ANOVA with Tukey's multiple comparisons test.

Box boundaries are the 25th and 75th percentiles, the horizontal line across the box is the median, and the whiskers indicate the minimum and maximum values. N = 8-12 biologically independent samples per group (144 total). Source data are provided in the Source Data file.

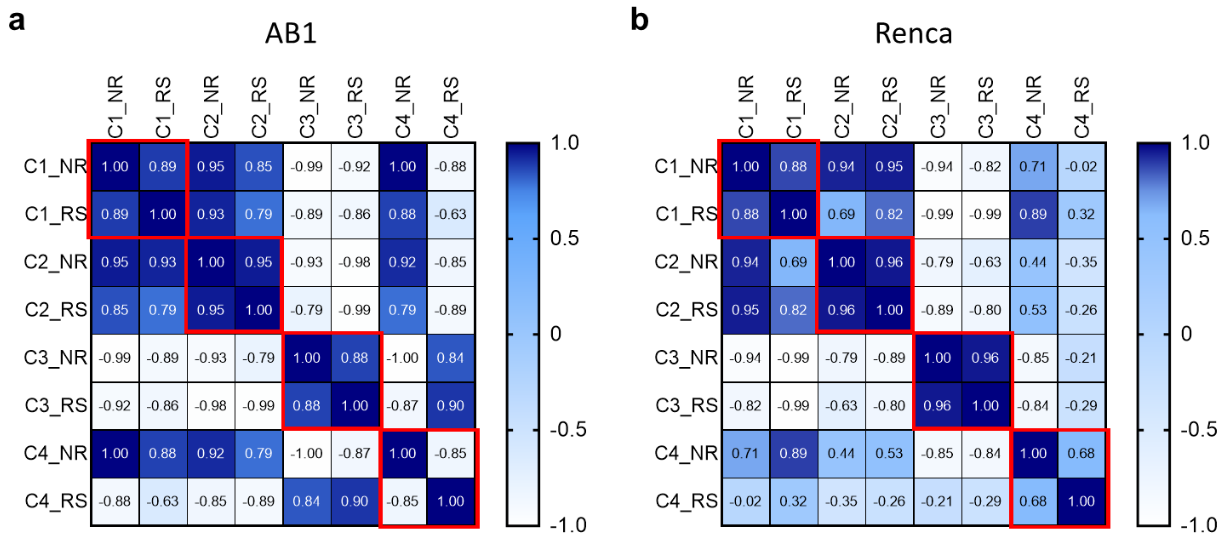

Supplementary Figure 3. Correlation analysis of the trendlines between responders and non-responders of each cluster identified by TCseq. (**a**) AB1 responders and non-responders have a similar trend (r>0.8) in clusters 1-3, but not in cluster 4. (**b**) Renca responders and non-responders have a similar trend (r>0.8) in clusters 1-3, but not in cluster 4. Source data are provided in the Source Data file.

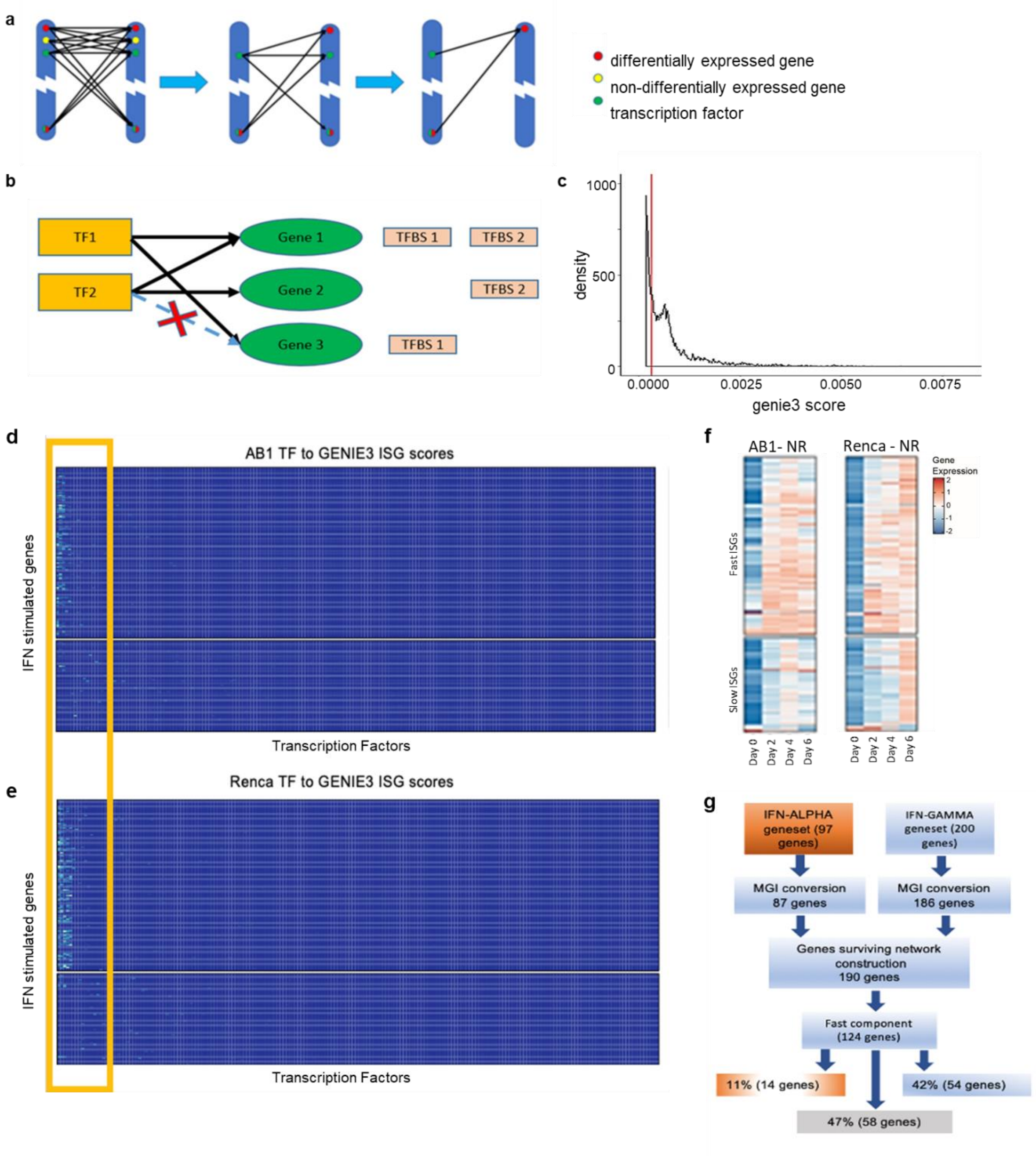

Supplementary Figure 4. Network construction approach and analysis on bulk RNA-seq data in AB1 and Renca. We constructed two "direct-interaction networks", one for AB1 and one for Renca using all responder samples for each strain across four experimental time points. (**a** and **b**) Pruning schema used to construct these networks. As illustrated in (b), we eliminate the GENIE3 edge from TF2 to differentially expressed gene 3 because gene 3 does not possess the transcription factor binding site for TF2. (**c**) Threshold selection after considering the union set of all GENIE3 weights from Renca and AB1 direct networks (red line - 0.0003125). (**d** and **e**) Full heatmap of TF to ISGs GENIE3 scores in AB1 (d) and Renca (e) from TFs (x-axis) to ISGs (y-axis). The yellow box across both heatmaps is displayed in Figure 2C, emphasizing that regulation is confined to IFN-related TFs. (**f**) Activation of interferon-stimulated genes over time in non-responders in AB1 (left) and Renca (right). (**g**) The on/fast-off component of ISGs is composed of genes stimulated by type I and type II IFN.

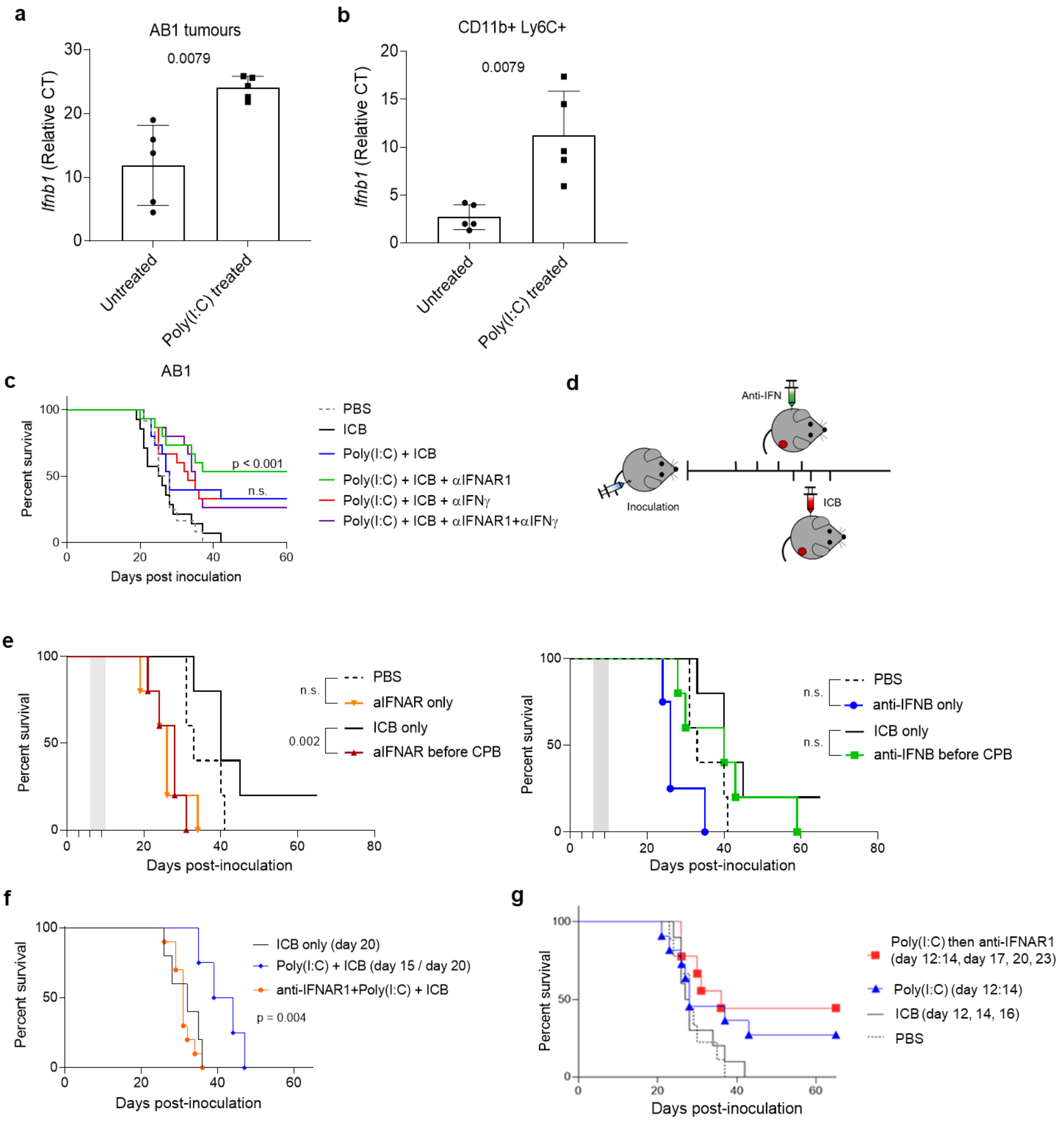

Supplementary Figure 5. Therapeutically phenocopying of on/fast-off type I IFN kinetics with poly(I:C) and anti-IFNAR improves response. (**a** and **b**) Poly(I:C) induces IFNβ production in Ly6C$^+$ monocytes in AB1 tumors. Tumors from AB1 bearing mice were treated with intratumoral poly(I:C), harvested, cell sorted, RNA extracted and analyzed for IFNβ expression by RT-PCR. (**a**) Relative IFNβ RNA expression in untreated compared to poly(I:C) treated tumors. (**b**) Relative IFNβ RNA expression within the sorted CD11b$^+$ Ly6C$^+$ monocyte population in untreated compared to poly(I:C) treated tumors. N = 5 biologically independent samples per group. Data are presented as mean values +/- SD. Source data are provided in the Source Data file. (**c**) Mice bearing AB1 tumors were treated with poly(I:C) (day 12, 13, 14), followed by ICB (day 17), followed by an antibody against IFNAR1, IFNγ, or both (day 20, 23, 26). Pre-treatment with poly(I:C) increases the number of responders, but does not significantly increase survival (blue line, p= 0.09, logrank test). Addition of anti-IFNAR significantly improves survival over ICB control

(green line, p = <0.001, logrank test). (**d**) Schedule of pre-treatment anti-IFNAR followed by ICB. (**e**) Dosing anti-IFNAR or anti-IFNβ alone is not significant compared to PBS controls. Dosing anti-IFNAR (but not anti-IFNβ) before ICB, rather than after, negates the effect of ICB on AE17 tumors (red line, p = 0.002, logrank test). (**f**) Dosing anti-IFNAR concurrently with poly(I:C) (orange line) negates the priming effect of poly(I:C) (p = 0.004, logrank test). (**g**) Blocking IFNAR after IFN stimulation with poly(I:C) does not affect response, even when ICB is not involved.

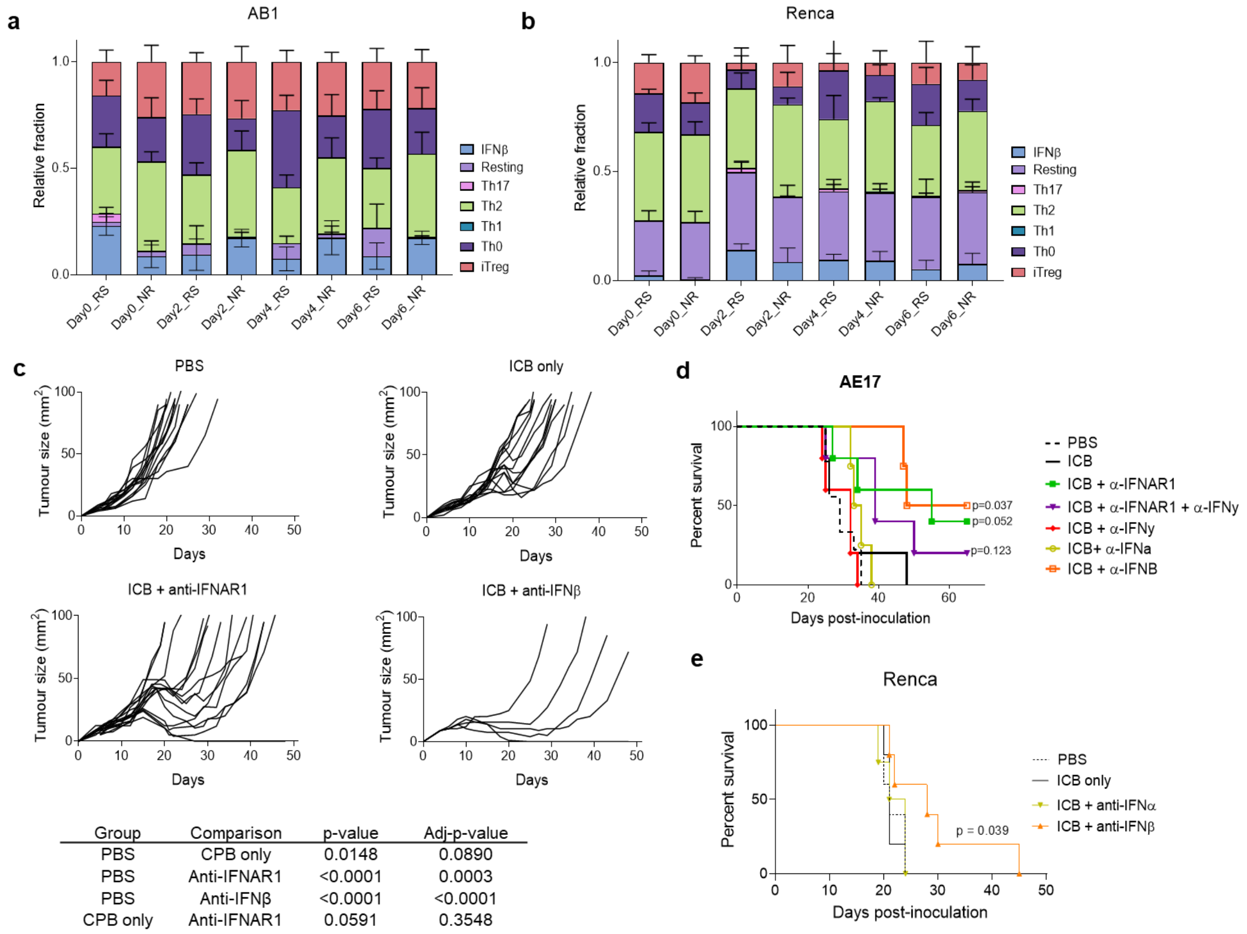

Group	Comparison	p-value	Adj-p-value
PBS	CPB only	0.0148	0.0890
PBS	Anti-IFNAR1	<0.0001	0.0003
PBS	Anti-IFNβ	<0.0001	<0.0001
CPB only	Anti-IFNAR1	0.0591	0.3548
CPB only	Anti-IFNβ	0.0032	0.0192
Anti-IFNAR1	Anti-IFNβ	0.0887	0.5324

Supplementary Figure 6. Mimicking IFN kinetics by blocking IFNβ after ICB improves responses.

(**a** and **b**) An IFNβ induced gene signature follows the on/off kinetics in AB1 and Renca tumors**.** Using a stimulated T cell gene expression signature from a recently published singe cell RNAseq dataset, which was obtained by treating human CD4+ T cells with chemokines including IFNβ *in vitro* [30], we constructed a reference matrix using CIBERSORT. Using this matrix, we used CIBERSORT to deconvolute these populations in our bulk RNAseq data for AB1 (**a**) and Renca (**b**). N = 8-12 biologically independent samples per group (72 per model). Error bars represent SD. The signature from the IFNβ T cells, but not other stimulated T cells, followed the rapid on/off kinetics in responders, but the increase is delayed in non-responders. Source data are provided in the Source Data file. (**c-e**) To more closely recapitulate a clinical scenario, pre-treatment with poly(I:C) was omitted from the schedule. (**c**) AB1 tumors treated with ICB followed by anti-IFNAR or anti-IFNβ showed a decrease in tumor size and delay in growth when complete response was not achieved. Growth curves were analyzed longitudinally using type II ANOVA and pairwise comparisons across groups with Bonferroni correction for multiple comparisons (lower table). (**d**) Blocking IFNβ, but not IFNα or IFNγ after ICB significantly improved response in the AE17 tumor model. (**e**) Blocking IFNβ, but not IFNα, after ICB significantly improved response in the Renca tumor model. (d) and (e) p-values are compared to ICB control, using Logrank testing.

a

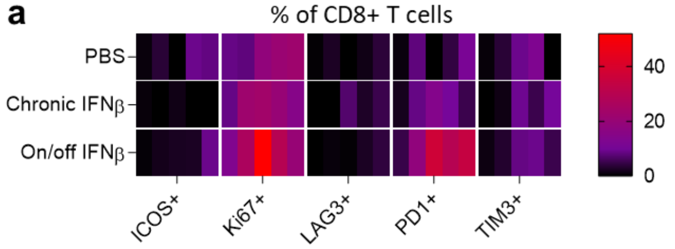

% of CD8+	ICOS	Ki67	LAG3	PD1	TIM3
PBS vs. Chronic IFNβ	0.740	0.912	0.964	0.834	1.000
PBS vs. On/off IFNβ	0.970	0.049	1.000	0.001	0.999
Chronic IFNβ vs. On/off IFNβ	0.869	0.124	0.960	0.006	0.999

b

% of CD45+

CD8+ % of CD45	ICOS	Ki67	LAG3	PD1	TIM3
PBS vs. Chronic IFNβ	0.987	0.901	0.975	0.940	0.999
PBS vs. On/off IFNβ	0.732	0.020	1.000	0.002	0.801
Chronic IFNβ vs. On/off IFNβ	0.636	0.061	0.974	0.005	0.830

c

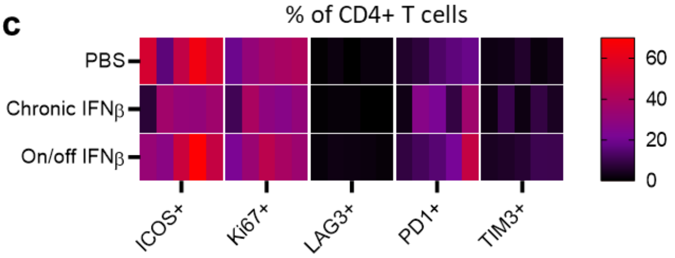

% of CD4+	ICOS	Ki67	LAG3	PD1	TIM3
PBS vs. Chronic IFNβ	0.003	0.535	0.994	0.412	0.905
PBS vs. On/off IFNβ	0.936	0.360	0.993	0.234	0.714
Chronic IFNβ vs. On/off IFNβ	0.001	0.951	0.975	0.930	0.932

d

% of CD45+

CD4+ % of CD45	ICOS	Ki67	LAG3	PD1	TIM3
PBS vs. Chronic IFNβ	0.438	0.801	0.999	0.999	0.993
PBS vs. On/off IFNβ	0.000	0.000	0.841	0.046	0.420
Chronic IFNβ vs. On/off IFNβ	0.000	0.000	0.818	0.051	0.487

e

CD4+ % of CD45	FoxP3+	FoxP3-
PBS vs. Chronic IFNβ	0.992	0.961
PBS vs. On/off IFNβ	0.302	0.098
Chronic IFNβ vs. On/off IFNβ	0.361	0.161

Supplementary Figure 7. Expression of immune checkpoints on tumour infiltrating T cells after chronic or on/off IFNB treatment. Mice bearing AE17 tumours were given recombinant IFNB i.t. daily for 6 days (chronic) or daily for 3 days followed by an anti-IFNB antibody (on/off). Tumours were harvested and analysed by flow cytometry. (**a**) Expression of immune checkpoints shown as % of $CD8^{+}$ T cells and associated p-values (**b**) P-values from comparisons of $CD8^{+}$ T cells expressing immune checkpoints as % of $CD45^{+}$ cells in Figure 4k. (**c**) Expression of immune checkpoints shown as % of $CD4^{+}$ T cells and associated p-values (**d**) P-values from comparisons of $CD4^{+}$ T cells expressing immune checkpoints as % of $CD45^{+}$ cells in Figure 4m. (**e**) P-values from comparisons of T-reg ($FoxP3^{+}$) or T-helper ($FoxP3^{-}$) $CD4^{+}$ T cells in Figure 4n. Tables show significance (adj. p-value) of comparisons from two-way ANOVA with Tukey's multiple comparisons test. (a) and (e) n = 5 biologically independent samples. Box boundaries are the 25th and 75th percentiles, the horizontal line across the box is the median, and the whiskers indicate the minimum and maximum values. P values calculated from two-way ANOVA with Tukey's multiple comparisons test. Source data are provided in the Source Data file.

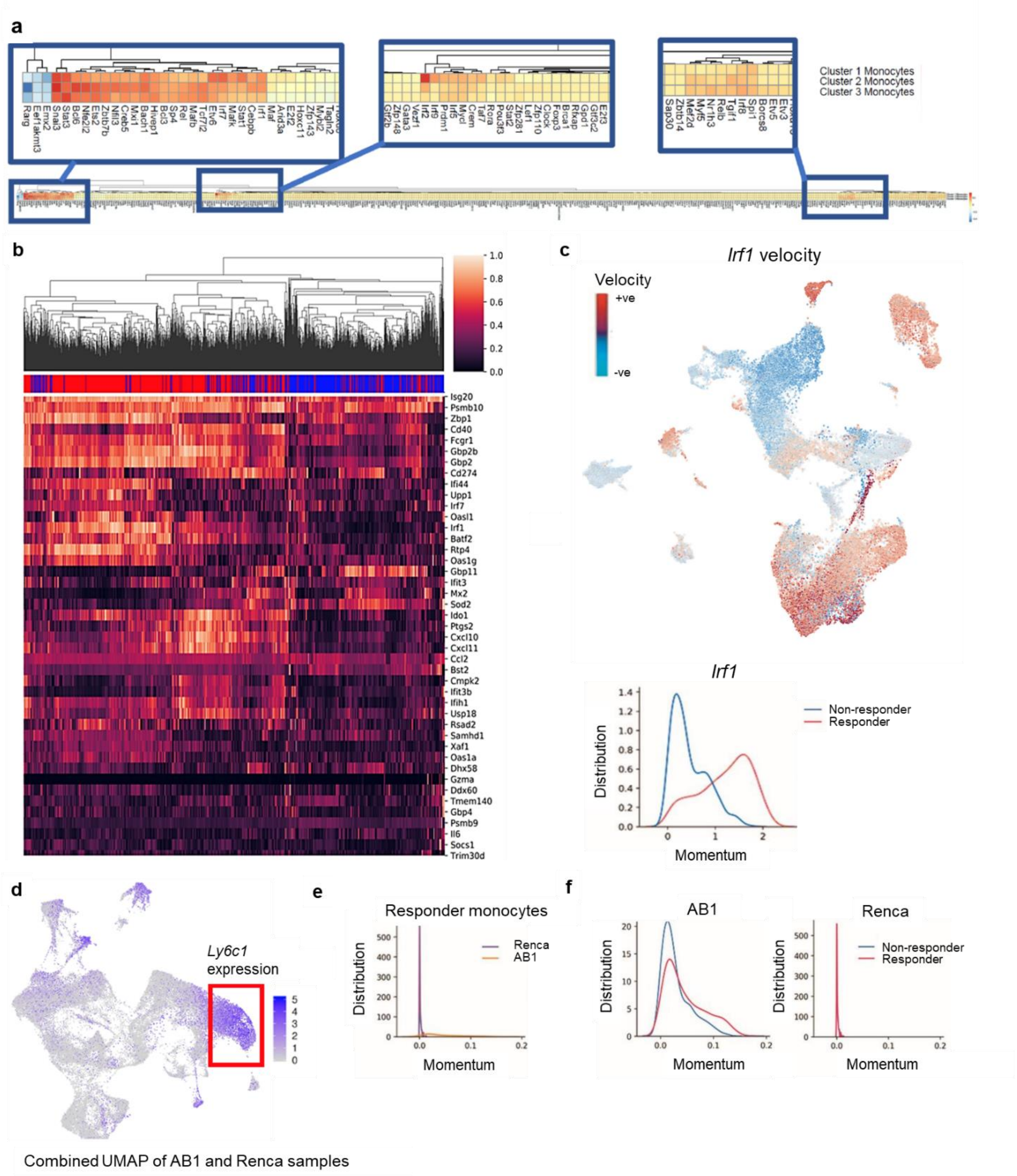

Supplementary Figure 8. Increased IFN-related activity in AB1 cluster 1 monocytes in responders, with Renca monocytes existing in an earlier transcriptional state. (**a**) SCENIC network analysis on single cell AB1 data showed differential transcription factor activation across the 3 monocyte populations. IFN-related TFs were found in all three regions of differential activation and were increased, especially in cluster 1 monocytes. (**b**) Heatmap of IFN-related gene transcriptional rates (abs(U_measured – U_predicted after gamma fit) for cluster 1 monocytes in responders (red) compared to non-responders (blue). (**c**) Irf1 transcription in single cells in AB1 tumors shows strong negative regulation in monocytes, and this is also more pronounced in re-

sponders than non-responders. (**d-f**) Since the bulk RNAseq data indicated that the Renca responder samples had a delayed on/fast-off dynamic compared to AB1, we queried whether this delayed dynamic in bulk RNAseq signal in Renca was due to an earlier state of the tumor microenvironment. To investigate this, we examined transcriptional activity of monocytes in AB1 and compared their activity to the same monocyte population in Renca. (**d**) Combined UMAP of AB1 and Renca samples with cluster 1 monocytes located in the region highlighted by the red box. In this region, Renca responder monocytes have reduced transcriptional momentum compared to AB1 monocytes (**e**). (**f**) Both responder and non-responder Renca monocytes have similar transcriptional momentum (right), unlike AB1 (left). Taken together, these data indicate that cluster 1 monocytes diverge over time along two transcriptional trajectories based on ICB response. These separate trajectories are characterised by differences in interferon gene expression, with Renca monocytes at an earlier transcriptional timepoint than AB1.

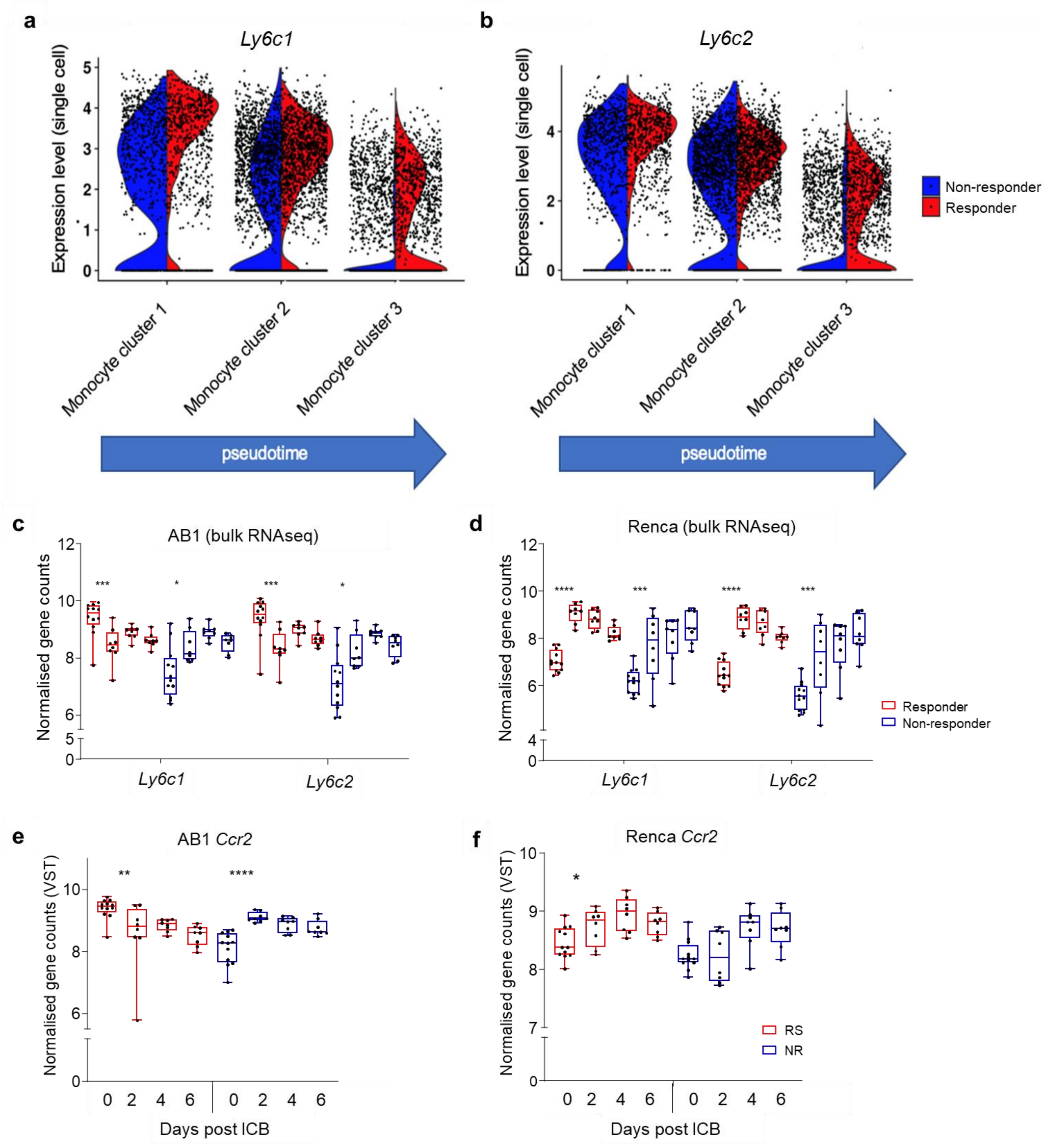

Supplementary Figure 9. On/off kinetics of Ly6c and CCR2 gene expression in responders over the course of ICB in bulk RNAseq data are compatible with Ly6c gene expression in responder AB1 monocytes along pseudo time axis.

(**a** and **b**) The kinetics of expression in *Ly6c1* (**a**) and *Ly6c2* (**b**) in AB1 monocyte clusters across the pseudo time axis (blue arrow, see also Figure 5H). (**c** and **d**) Box plot of the normalized expression of the genes *Ly6c1* and *Ly6c2* in AB1 (**c**) and Renca (**d**) bulk RNAseq data. Expression is plotted over time (left to right) in responders (red) and non-responders (blue). (**e** and **f**) Box plot of the normalized expression of *Ccr2* in AB1 (**e**) and Renca (**f**) over time. Comparisons are between day 0 and day 2 within the same group. N = 8-12 biologically independent samples (n = 72 total per model). Box boundaries are the 25th and 75th percentiles, the horizontal line

across the box is the median, and the whiskers indicate the minimum and maximum values. p ≤ 0.05, **p ≤ 0.01, ****p ≤ 0.001 , ****p < 0.0001 from two-way ANOVA with Tukey's multiple comparisons test. Source data are provided in the Source Data file.

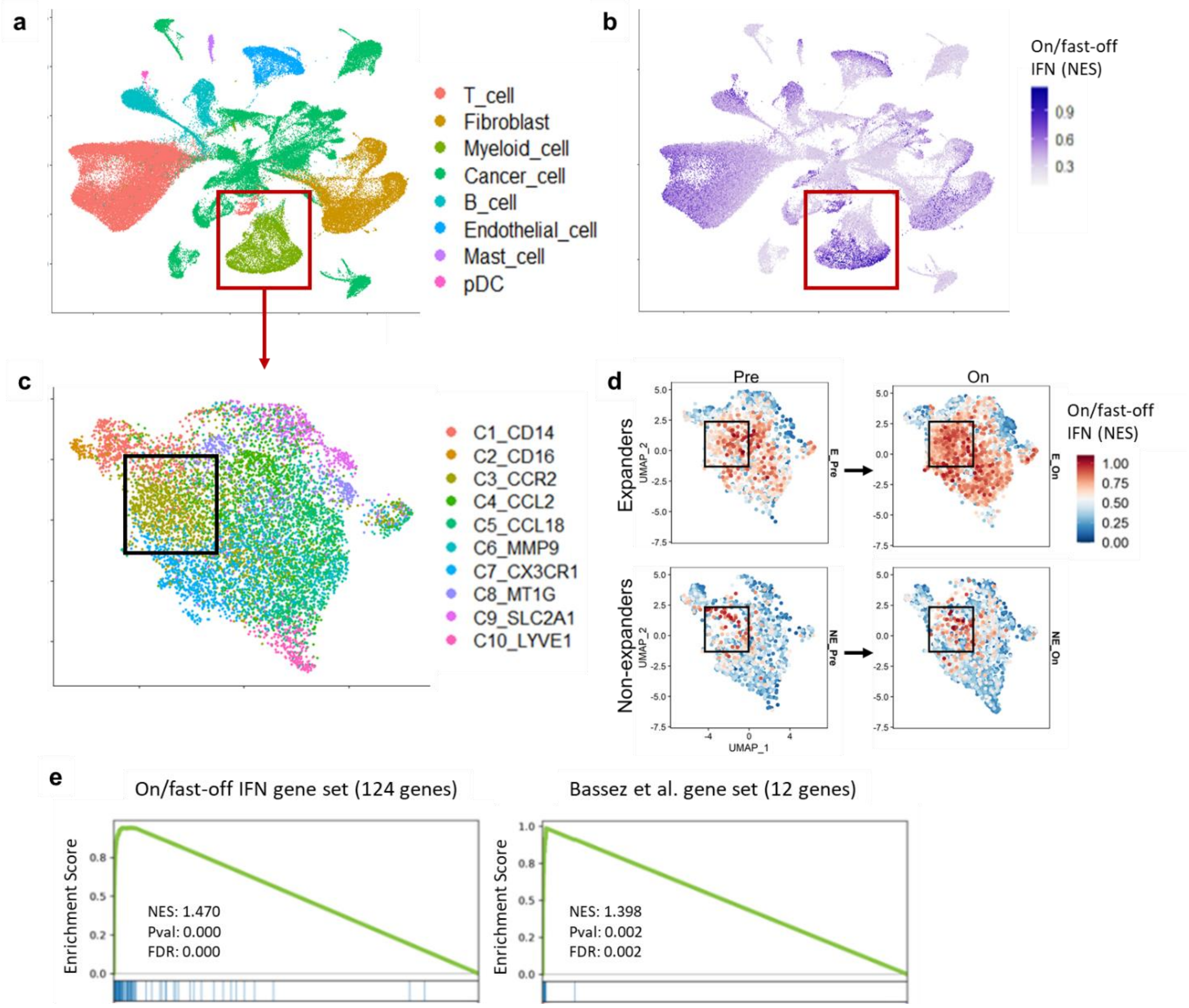

Supplementary Figure 10. Patients treated with ICB display early enrichment of the on/fast-off IFN signature.

(**a**) UMAP of single cells from breast cancer patient biopsies taken prior to treatment with anti-PD1. Cluster labels have been provided from the original published analysis [25]. (**b**) UMAP showing a highest enrichment of the on/fast-off IFN signature within the myeloid cluster. (**c**) UMAP of the myeloid cell subset from (a), with cell labels provided from the original published analysis. (**d**) UMAP of myeloid cells in expander and non-expanders, showing highest enrichment in the CCR2 population. (**e**) Pre-ranked gene set enrichment analysis of expander vs non-expander CCR2^{+} cells using the on/fast-off-IFN gene signature or the IFN gene signature published with the breast cancer patient dataset. (NES = normalized enrichment score)

SUPPLEMENTARY METHODS

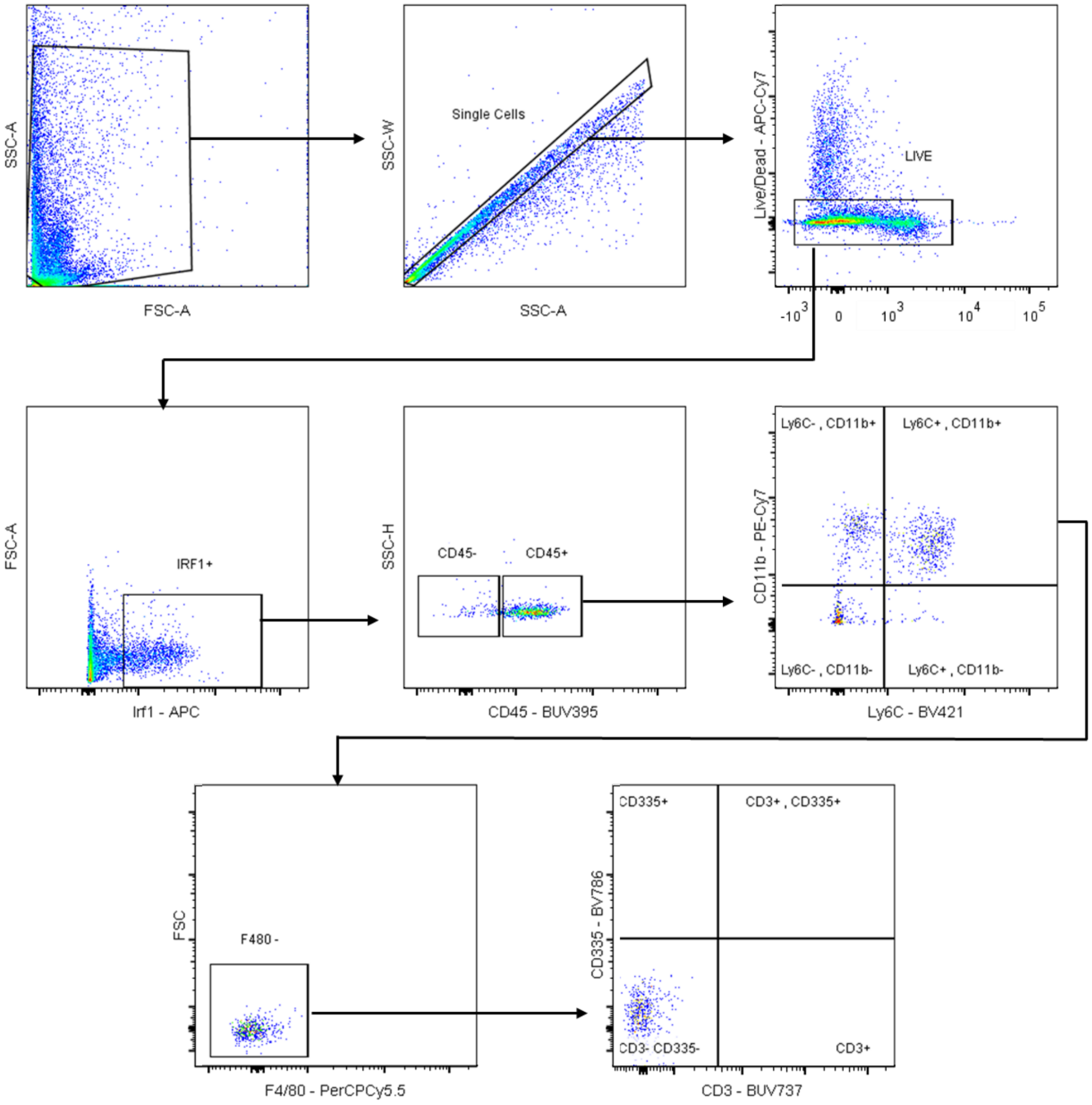

Supplementary Figure 11. Flow cytometry gating strategy of *Irf1*$^{+}$ cell markers.

Cells were stained using RNA target probes for *Irf1* RNA. Irf1^{+} cells were then gated to determine the phenotype of the cells. CD45 expression was used to distinguish immune cells: CD45^{-} non-immune cells (e.g. tumor cells) and CD45^{+} immune cell populations. Immune cells were analyzed by their expression of CD11b and Ly6C. Ly6C^{+} CD11b^{+} immune cells were F4/80^{-}, CD3^{-} and CD335^{-}.

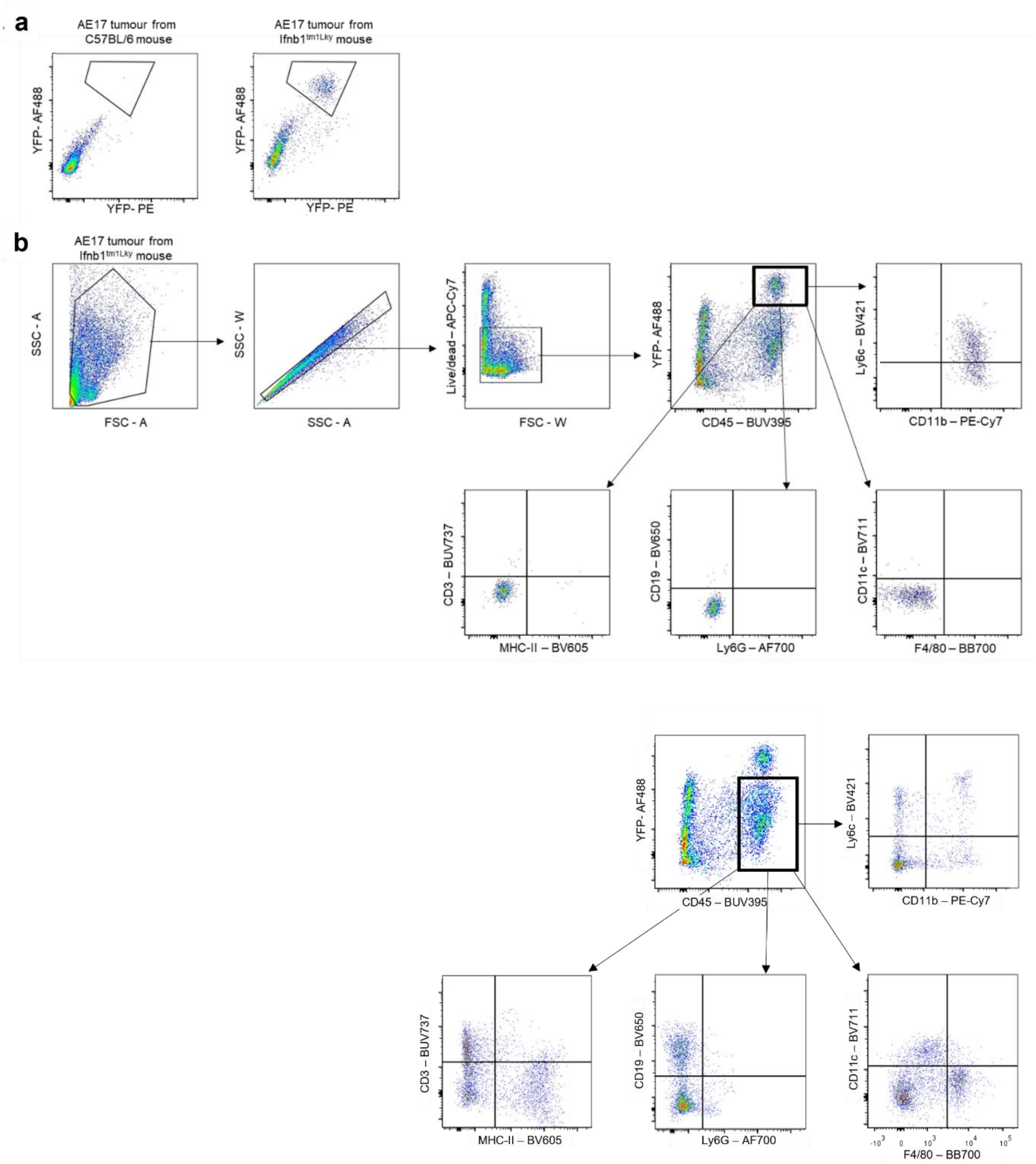

Supplementary Figure 12. Flow cytometry gating strategy of YFP^{+} cell markers.

(**a**) Cells were stained using an anti-GFP antibody to detect YFP^{+} cells. AE17 tumours from C57BL/6 wildtype mice were used as a negative control. (**b**) Single cells that were negative for our dead cell marker were gated. YFP^{+} cells were exclusively CD45^{+} (immune cells) and then were gated to determine the phenotype of the cells. YFP^{+} immune cells were CD11b^{+}, Ly6C+/-, MHC-II^{-}, CD11c^{-}, Ly6G^{-}, CD19^{-}, CD3^{-} and F4/80^{-}. Phenotype of YFP^{-} cells are given as a comparison.

Supplementary Table 1. Flow cytometry antibodies.

Antibody	Fluorophore	Clone	Vendor	Cat #	Dilution
CD45	BUV395	30-F11	BD	564279	1:1000
CD3	BUV737	17A2	BD	741788	1:200
Ly6C	BV421	AL-21	BD	562727	1:200
CD335 (NKp46)	BV786	29A1.4	BD	741029	1:200
F4/80	BB700 or APC	T45-2342	BD	746070	1:500
CD11b	PE-Cy7	M1/70	BD	561098	1:200
CCR2	BV650	475301	BD	747968	1:200
Fixable Viability Stain 780			BD	565388	1:2000
Type 1 Mouse Irf1 RNA Target Probe Set	Alexa Fluor 647	VB1-3028161-PF	Invitrogen	8818005-210	
MHC-II (I-A/I-E)	BV605	M5/114.15.2	BD	563413	1:500
CD11c	BV711	HL3	BD	563048	1:200
Ly6G	AF700	1A8	BD	561236	1:500
CD19	BV650	1D3	BD	563235	1:200
CD8a	BV480	53-6.7	BD	566169	1:200
CD4	BUV496	GK1.5	BD	612952	1:200
GFP (YFP-cross reactive polyclonal)	AF488	A-21311	Thermo-Fisher	A-21311	1:200
GFP (YFP-cross reactive monoclonal)	PE	FM264G	BioLegend	338003	1:200
PD1	BV421	J43	BD	562584	1:200
TIM3	BV650	RMT3-23	BD	747623	1:100
LAG3	BB700	C9B7W	BD	742206	1:100
ICOS	PE	7E.17G9	BioLegend	117405	1:200
Ki67	AF700	B56	BD	561277	1:200
FoxP3	APC	MF23	BD	560402	1:200
