# Supplementary material for: Temporally restricted activation of IFNβ signaling underlies response to immune checkpoint therapy in mice
Source: Nat Commun. 2022 Aug 19;13:4895. doi: 10.1038/s41467-022-32567-8 (PMC9390963; doi:10.1038/s41467-022-32567-8)
Supplement: Supplementary file 3 — Description of Additional Supplementary Files [file 41467_2022_32567_MOESM3_ESM.docx]

**Supplementary Data 1**. Differentially expressed genes between responders and non-responders in bulk RNAseq data in AB1 and Renca mice

**Supplementary Data 2**. CIBERSORT CD8+ T cell subset comparisons

**Supplementary Data 3**. Gene lists for each TCseq cluster

**Supplementary Data 4**. Genes comprising each module for diagram in Figure 3A and enrichment analysis

**Supplementary Data 5**. On/fast-off IFN gene set with human orthologs

**Supplementary Data 6.** Proportion of all cell types form the AB1 single cell data

**Supplementary Data 7.** Preranked gsea analysis and Pagoda2 overdispersion analysis in murine and human single cell datasets

**Supplementary Data 8**. CIBERSORT stimulated T cell reference matrix
