# Supplementary material for: Temporally restricted activation of IFNβ signaling underlies response to immune checkpoint therapy in mice
Source: Nat Commun. 2022 Aug 19;13:4895. doi: 10.1038/s41467-022-32567-8 (PMC9390963; doi:10.1038/s41467-022-32567-8)
Supplement: Supplementary file 12 — Reporting Summary [file 41467_2022_32567_MOESM12_ESM.pdf]

nature portfolio

Corresponding author(s): W. Joost Lesterhuis
Timo Lassmann

Last updated by author(s): Jun 15, 2022

Reporting Summary

Nature Portfolio wishes to improve the reproducibility of the work that we publish. This form provides structure for consistency and transparency in reporting. For further information on Nature Portfolio policies, see our Editorial Policies and the Editorial Policy Checklist.

Statistics

For all statistical analyses, confirm that the following items are present in the figure legend, table legend, main text, or Methods section.

n/a	Confirmed	
☐	☒	The exact sample size (*n*) for each experimental group/condition, given as a discrete number and unit of measurement
☐	☒	A statement on whether measurements were taken from distinct samples or whether the same sample was measured repeatedly
☐	☒	The statistical test(s) used AND whether they are one- or two-sided<br>*Only common tests should be described solely by name; describe more complex techniques in the Methods section.*
☐	☒	A description of all covariates tested
☐	☒	A description of any assumptions or corrections, such as tests of normality and adjustment for multiple comparisons
☐	☒	A full description of the statistical parameters including central tendency (e.g. means) or other basic estimates (e.g. regression coefficient) AND variation (e.g. standard deviation) or associated estimates of uncertainty (e.g. confidence intervals)
☐	☒	For null hypothesis testing, the test statistic (e.g. *F*, *t*, *r*) with confidence intervals, effect sizes, degrees of freedom and *P* value noted<br>*Give P values as exact values whenever suitable.*
☒	☐	For Bayesian analysis, information on the choice of priors and Markov chain Monte Carlo settings
☒	☐	For hierarchical and complex designs, identification of the appropriate level for tests and full reporting of outcomes
☐	☒	Estimates of effect sizes (e.g. Cohen's *d*, Pearson's *r*), indicating how they were calculated

Our web collection on statistics for biologists contains articles on many of the points above.

Software and code

Policy information about availability of computer code

Data collection	Flow cytometric data collected using BD FacsDiva<br>We processed single cell samples using the cellranger3 software from 10X genomics
Data analysis	Code for the analysis in the main manuscript and the extended data is available through GitHub (https://github.com/melvinchin/dynamic_interferon_signalling)<br>Programs/packages used:<br>R studio (v3.3.3)<br>FastQC (v0.11.3)<br>Kallisto (v0.43.0)<br>Sleuth (v0.29.0)<br>GENIE3 (v1.8.0)<br>igraph package (v1.2.4.2)<br>HiveR package (v0.3.42)<br>ggplot2 package (v3.2.1)<br>ComplexHeatmap42 package (v2.2.0).<br>Seurat R package (v3.0.3)<br>SingleR (v1.0.5)<br>Other R packages; R data.table, inferCNV, AUCell, scde, EnrichR, Pheatmap, Cellrouter, phenopath<br>velocyto.py package (v0.17.16)<br>Loompy (v3.0.0).<br>seaborn<br>CIBERSORTx- estimate the relative proportions of immune cell types based on the transcriptomic profiles (https://cibersortx.stanford.edu/)

GraphPad PRISM (v8) - graphical and statistical program
FlowJo (v10) - flow cytometric analysis
BD FacsDiva (v9.0)
cellranger (v3)- single cell RNA data

For manuscripts utilizing custom algorithms or software that are central to the research but not yet described in published literature, software must be made available to editors and reviewers. We strongly encourage code deposition in a community repository (e.g. GitHub). See the Nature Portfolio guidelines for submitting code & software for further information.

Data

Policy information about availability of data

All manuscripts must include a data availability statement. This statement should provide the following information, where applicable:

- Accession codes, unique identifiers, or web links for publicly available datasets
- A description of any restrictions on data availability
- For clinical datasets or third party data, please ensure that the statement adheres to our policy

The RNA-Seq generated in this study have been deposited in the Gene Expression Omnibus database under accession code GSE153941 (https://www.ncbi.nlm.nih.gov/geo/query/acc.cgi?acc=GSE153941) for the bulk RNAseq data, and GEO: GSE153942 (https://www.ncbi.nlm.nih.gov/geo/query/acc.cgi?acc=GSE153942) for the single cell RNAseq data. The human breast cancer single cell data (https://doi.org/10.1038/s41591-021-01323-8) used in this study is publicly available to download as read count data per individual patient at http://biokey.lambrechtslab.org.
Source data for graphs are provided with this paper.
Code for the analysis in the main manuscript and the supplementary data is available through GitHub (https://github.com/wlchin/IFNsignalling).

Field-specific reporting

Please select the one below that is the best fit for your research. If you are not sure, read the appropriate sections before making your selection.

[x] Life sciences [ ] Behavioural & social sciences [ ] Ecological, evolutionary & environmental sciences

For a reference copy of the document with all sections, see nature.com/documents/nr-reporting-summary-flat.pdf

Life sciences study design

All studies must disclose on these points even when the disclosure is negative.

Sample size	RNAseq: The sample size for the bulk RNAseq experiments was estimated using the method developed by Hart et al; for sample sizes of n=12 and a within group coefficient of variation of 0.3 there is >90% power to detect a 1.5-fold change in gene expression, for sample sizes of n=8 and a within group coefficient of variation of 0.24 there is >90% power to detect a 1.5-fold change in gene expression.<br>Mouse experiments: The sample size calculation for in vivo mouse experiments was based on prior experiments in which we found that the median survival time on the control treatment (ICB alone) was 35 days. Using a proportional hazards model we determined that, if the true hazard ratio (relative risk) of control subjects relative to experimental subjects is 5, we would need to study 10 experimental subjects and 10 control subjects to be able to reject the null hypothesis that the experimental and control survival curves are equal with probability (power) 0.8. The type I error probability associated with this test of this null hypothesis is 0.05.<br>For single cell RNAseq, 3 responder and 3 non-responder samples were used for each model, with the aim to collect 6000 cells per sample. No sample size calculation was performed for this experiment, as it was used to identify which cell population expressed the IFN signature, therefore 3 samples per group was used to limit the effect of spurious variation and capture sufficient cells.<br>For experiments used to confirm our findings (PCR, flow cytometry), we have found that 5 biological replicates per group is enough to assess and isolate sources of variation in measurements and limit the effect of spurious variation.
Data exclusions	For RNA sequencing, "intermediate responders"- mice that had a partial response to therapy, were excluded from analysis as they did not reach the pre-established criteria for "responder" (complete response) or "non-responder" (no response), and therefore those samples were not processed. No data were excluded from computational analyses. All filtering has been defined.
Replication	For the bulk RNAseq, 144 biologically independent samples were from 18 independent experiments. All of these experiments successfully replicated the finding that there were responder and non-responders within an experiment.<br>Experimental results shown are from 2 or 3 experiments per murine model, and most are repeated in at least two different murine tumour models. All attempts at replication were successful.
Randomization	Age-matched mice after inoculation with tumour cells (by investigator 1) were randomised between cages (by investigator 2). Cages were then randomly assigned an experimental group.<br>RNA samples were dispersed across several lanes (1 sample from each group and time point for 8 samples per lane, over 9 lanes per model) to avoid run-bias during sequencing.

Blinding	Investigators were blinded to experimental groups during data collection. For murine experiments, one investigator would administer treatment, and a blinded investigator would collect data on tumour growth for analysis. The investigator administering treatment would be blinded to the tumour growth results until the experiment was complete, including sample harvest, acquisition and analysis. For RNAseq experiments, investigators had to determine which samples were from responder or non-responder mice, to allocate samples for sequencing. Investigators needed to know which groups the samples belonged to to complete the RNAseq analysis (eg. differential expression, time-course analysis).

Reporting for specific materials, systems and methods

We require information from authors about some types of materials, experimental systems and methods used in many studies. Here, indicate whether each material, system or method listed is relevant to your study. If you are not sure if a list item applies to your research, read the appropriate section before selecting a response.

Materials & experimental systems

n/a	Involved in the study	
☐	☒	Antibodies
☐	☒	Eukaryotic cell lines
☒	☐	Palaeontology and archaeology
☐	☒	Animals and other organisms
☒	☐	Human research participants
☒	☐	Clinical data
☒	☐	Dual use research of concern

Methods

n/a	Involved in the study	
☒	☐	ChIP-seq
☐	☒	Flow cytometry
☒	☐	MRI-based neuroimaging

Antibodies

Antibodies used

Flow cytometry
Antibody / Fluorophore / Clone / Vendor / Cat # / Dilution
CD45 BUV395 30-F11 BD 564279 1:1000
CD3 BUV737 17A2 BD 741788 1:200
Ly6C BV421 AL-21 BD 562727 1:200
CD335 (NKp46) BV786 29A1.4 BD 741029 1:200
F4/80 BB700 or APC T45-2342 BD 746070 1:500
CD11b PE-Cy7 M1/70 BD 561098 1:200
CCR2 BV650 475301 BD 747968 1:200
Fixable Viability Stain 780 BD 565388 1:2000
MHC-II (I-A/I-E) BV605 M5/114.15.2 BD 563413 1:500
CD11c BV711 HL3 BD 563048 1:200
Ly6G AF700 1A8 BD 561236 1:500
CD19 BV650 1D3 BD 563235 1:200
CD8a BV480 53-6.7 BD 566169 1:200
CD4 BUV496 GK1.5 BD 612952 1:200
GFP (YFP-cross reactive polyclonal) AF488 A-21311 Thermo-Fisher A-21311 1:200
GFP (YFP-cross reactive monoclonal) PE FM264G BioLegend 338003 1:200
PD1 BV421 J43 BD 562584 1:200
TIM3 BV650 RMT3-23 BD 747623 1:100
LAG3 BB700 C9B7W BD 742206 1:100
ICOS PE 7E.17G9 BioLegend 117405 1:200
Ki67 AF700 B56 BD 561277 1:200
FoxP3 APC MF23 BD 560402 1:200

PrimeFlow
Type 1 Mouse Irf1 RNA Alexa Fluor 647 Target Probe Set, BD (VB1-3028161-PF, cat. PF-204)

in vivo
anti-PD-L1, clone MIH5, Bioceros
anti-CLTA4, clone 9H10, Bioceros
anti-PD-L1, clone MIH5, Bioxcell
anti-CLTA4, clone 9H10, Bioxcell
anti-IFNAR1 clone MAR1-5A3, Bioxcell
anti-IFNγ, clone XMG1.2, Bioxcell
anti-IFNα, clone TIF-3C5, Leinco
anti-IFNβ, clone HDß-4A7, Leinco
IgG2a isotype, clone C1.18.4, Leinco

Validation

Antibodies were tested on the intended tissues (mouse tumour) using standard protocols as suggested by the manufacturer. All

antibodies were tested on fresh tissue. Antibodies for intracellular GFP, ICOS, Ki67, FoxP3 and LAG3 were tested on fixed tissue using a cytofix/cytoperm kit (cat # 554714, BD). 5 dilutions were tested using the manufacturers recommendation as a guide. PrimeFlow was done as per manufacturer's instructions and recommendations. anti-IFNAR1, anti-IFNy, anti-IFNa and anti-IFNB have been tested on mouse cell lines stimulated with poly(I:C) to assess the up-regulation of receptors associated with IFN signalling by flow cytometry. In vivo checkpoint antibodies have been tested by our lab previously for the optimal dosing schedule (anti-CTLA4 100ug, one dose + anti-PD-L1 100ug, given every 2nd day for a total of 3 doses).

Eukaryotic cell lines

Policy information about cell lines

Cell line source(s)	AB1- CellBank Australia<br>Renca- donated by Dr E. Sotomayor and Dr F. Cheng (University of South Florida, Tampa, FL). Cells are from the ATCC, USA.<br>AE17- CellBank Australia
Authentication	Cell lines were validated yearly by flow cytometry for MHC class I molecules H2-Kb (consistent with C57BL/6) and H2-Kd (consistent with BALB/c), and for fibroblast markers E-cad, EpCam and PDGFRa (negative) and by PCR for mesothelin (positive for AB1, negative for Renca).
Mycoplasma contamination	Cells were frequently tested for mycoplasma by PCR and remained negative
Commonly misidentified lines (See ICLAC register)	In this study no commonly misidentified cell lines were used

Animals and other organisms

Policy information about studies involving animals; ARRIVE guidelines recommended for reporting animal research

Laboratory animals	Mus musculus strains BALB/cArc, BALB/cJAusb and C57BL6/J were used in this study. Mice were all female, at 8-12 weeks of age. Female Ifnb1tm1Lky/J mice were used at 6-10 weeks of age. Mice were housed at 21-22°C, 60% humidity with 12-hour light/dark cycle (06:00 – 18:00). Mice were fed Rat and Mouse cubes (Specialty Feeds, Glen Forrest, Australia) and had access to water ad libitum. Cages (Techniplast, Italy) were individually ventilated with filtered air, contained aspen chips bedding (Tapvei, Estonia) and were supplemented with tissues, cardboard rolls and wood blocks as environmental enrichment, and were changed every 14 days.
Wild animals	This study did not involve wild animals
Field-collected samples	This study did not involve field-collected samples
Ethics oversight	All experiments were approved by the Harry Perkins Institute for Medical Research animal ethics committee (approval numbers AE047, AE091, AE157).

Note that full information on the approval of the study protocol must also be provided in the manuscript.

Flow Cytometry

Plots

Confirm that:

- [x] The axis labels state the marker and fluorochrome used (e.g. CD4-FITC).
- [x] The axis scales are clearly visible. Include numbers along axes only for bottom left plot of group (a 'group' is an analysis of identical markers).
- [x] All plots are contour plots with outliers or pseudocolor plots.
- [x] A numerical value for number of cells or percentage (with statistics) is provided.

Methodology

Sample preparation	AB1 tumours were harvested 6 days after inoculation and immediately submerged in cold PBS, cut into 1-2 mm pieces with a scalpel blade and dissociated using the GentleMACS system (Miltenyi). Fc block (anti-CD16/CD32, BD) was used for 10 minutes on ice. Cell were stained with UV Zombie live/dead (Biolegend) for 30 minutes at RT, to discriminate live cells. Cells were stained using antibodies for surface markers for 30 minutes at 4 degrees. To identify Irf1+ cells, we used the PrimeFlow Kit (Invitrogen).
Instrument	BD LSR Fotessa flow cytometer X-20<br>Cell sorting: BD Influx
Software	Collection: FacsDiva<br>Analysis: FlowJo, PRISM
Cell population abundance	Cell sort: 100,000 to 200,000 cells collected. non-immune cells (CD45-) ~62% ; Ly6Chi monocytes (CD45+ CD11b+ Ly6Chi CD3- CD335-) ~11%, Ly6Clo/- monocytes (CD45+ CD11b+ Ly6Clo/- CD3- CD335-) ~14%, and the remaining immune cell (CD45

+CD11b-) ~13%.

Gating strategy	Gating strategy available in Supplementary Information<br>Gates were determined by use of fluorescence minus one controls

☒ Tick this box to confirm that a figure exemplifying the gating strategy is provided in the Supplementary Information.
